# An Index of Treatment

**Published:** 1915-12

**Authors:** 


**An Index of Treatment.** By Various Writers. Edited by ROBERT HUTCHISON, M.D., F.R.C.P., and JAMES SHERREN, F.R.C.S. Seventh Edition. Pp. xvi. 1,143. 1915. Bristol: John Wright & Sons Ltd. [Price 21s.]

The first edition of this work, published in 1907, was intended to provide the practitioner with a complete guide to treatment in moderate compass, and in a form convenient for reference. That it has already reached the seventh edition in eight years shows that the authors have been successful in their endeavours. Several new articles have been added, amongst them being those on Sterility, Radium-therapy, and the Psychoneurosis; seven pages on this last difficult topic give an excellent outline of the methods of treatment employed by Freud and his school.

The book is the combined effort of some ninety authors, who are leading authorities on their respective topics. It is well printed, well bound, has a supplementary index of forty-two closely-printed pages, and cannot fail to be of great service to those who will keep it on the table for ready reference.

